# Early Specialization and Progress of Finalist Swimmers in World Championships and Olympic Games

**DOI:** 10.3390/jfmk9040187

**Published:** 2024-10-07

**Authors:** Inmaculada Yustres Amores, Jesús Santos del Cerro, Víctor Rodrigo Carranza, Francisco Hermosilla-Perona

**Affiliations:** 1 Facultad de Ciencias de la Salud, Universidad Francisco de Vitoria, Ctra. Pozuelo-Majadahonda, Km 1.800, Pozuelo de Alarcón, 28223 Madrid, Spain; 2 Department of Economics, University of Castilla-La Mancha, San Pedro Mártir, 45004 Toledo, Spain; 3 Sport Training Lab, University of Castilla-La Mancha, 45008 Toledo, Spain; victor.rodrigo@uclm.es; 4 Facultad de Ciencias de la Vida y la Naturaleza, Universidad Nebrija, 28248 Madrid, Spain; hermosilla1995@gmail.com; 5 Department of Physical Activity and Sports Science, Alfonso X El Sabio University, 28691 Madrid, Spain

**Keywords:** youth, performance, talent, competition, analysis

## Abstract

**Background:** The main objectives of this study were to analyze the effect of early specialization in swimming and to observe the general patterns of success of two different sport specialization models [Spanish (SPA) and United States of American (USA) swimmers] participating in World Championships (WCs) and Olympic Games (OGs) between the years 2006 and 2021 of all swimming strokes and distances. **Methods:** Descriptive analyses and contingency tables were examined for all the variables. Explanatory models of the z scores were estimated from age depending on the events’ distances and strokes. Quadratic regression models were developed to capture the behavior of the variable time with parabolic functions, and the significance of the global model and the predictor variables (age) were also evaluated. In addition, the optimal age (peak performance) as well as the curvature of the model were analyzed. These models were compared between SPA and USA swimmers. **Results:** The results showed that the main differences in the patterns to success between SPA and USA were the earlier participation of USA swimmers in high-level competitions, as well as the greater number of participants for all the strokes, events, genders, and competitions. Age peak performance in short distances was lower for Spanish swimmers, obtaining the opposite situation for long distances. **Conclusions:** Being a finalist in junior WCs did not influence success in the finals of the senior WCs and OGs. Main differences in general patterns of success between SPA and USA showed younger swimmers participating in short-distance events, backstroke, and butterfly for USA swimmers and older swimmers taking part in butterfly and short-distance events for SPA.

## 1. Introduction

The World Championships (WCs) and Olympic Games (OGs) are the benchmark events for athletes of all disciplines from all over the world. More specifically, the swimming performance achieved in these events has increased significantly in recent years [1], which is mainly associated with improvements in biological, cognitive, and training factors [2,3,4].

In recent years, the early specialization or the performance achieved in the junior category has aroused much interest [5,6,7] since it has been questioned as a determining factor for their subsequent achievement performance in senior ages. Early specialization can be defined as the achievement of four specific parameters: Ref. [1] stated these as an early age of starting in the sport; early participation in a sport (as opposed to participating in multiple sports); early participation in an intensive training process; and early participation in competitive sport (participation in WCs and OGs in this investigation) [5].

Today, there is a trend towards early specialization in sports [2,5,7,8,9]. Furthermore, appended to the early specialization, another aspect that should be considered is the international debut of those swimmers who achieve a great performance in the early stages [8]. In this sense, studies conclude that having an early specialization does not have a positive relationship with exhaustion and abandonment of swimming in some specific samples [10], although some young athletes who seem to be future promises fail at the sporting level [11]. On the other hand, it has been seen that, in swimmers who have an excellent performance as a junior, when they reach the senior category, they have a greater probability of success in the WCs [12]. Thus, being able to participate in the junior WCs can lead to success in the senior WCs [13]. So, the countries with swimmers who achieved optimal performance in the junior WCs also had more possibilities to reach a top position at the senior WCs [14].

Historically, the United States of America (USA) has been one of the great favorites in all swimming competitions. Also, it is the country that has achieved the highest number of medals in the history of swimming in the senior stage at the WCs and at the same age in the OGs [15]. The U.S. swimming system emphasizes a balanced, long-term athlete development with late specialization, while Spain focuses on early specialization through intense training in clubs from a young age. Both systems have distinct strengths and challenges regarding athletes’ well-being and performance. Previous researchers have suggested that this may be due to the sports specialization model based on achieving performance in college leagues before turning professional [16]. On the other hand, other European countries (such as SPA) do not have a strong university sports system, and the social and economic recognition of university sports is not as strong as in the USA. This leads to different patterns of early specialization. However, the level of European countries such as SPA in the WCs and OGs has been growing in recent years. Thus, the main objective of this study was to analyze the effect of early specialization in swimming of two different early specialization systems (USA vs. SPA). In addition, another objective was to observe the general patterns of the success of SPA and USA swimmers participating in the Olympic Games and World Championships between the years 2006 and 2021 for all strokes and distances.

## 2. Materials and Methods

The present study is an original observational retrospective study. Due to the progress made by the swimmers who participated in the junior category and later in the senior category in the WCs and OGs in swimming was analyzed in order to identify different trends between USA and SPA. The data analyzed corresponds to the finalist swimmers of the WCs and OGs, from 2006 to 2021, of SPA and USA swimmers as the first junior WCs were carried out in 2006. The data were found on the page of the International Swimming Federation (FINA) (https://www.fina.org/, accessed on 2 July 2022), and the swimmers were divided into junior and senior categories, with junior WCs taking place in 2006, 2008, 2009, 2011, 2013, 2015, 2017, and 2019, senior WCs in 2007, 2009, 2011, 2013, 2015, 2017, and 2019, and the Olympic Games in 2008, 2012, 2016, and 2021.

### 2.1. Subjects

The database initially contained a total of 631 entries corresponding to a total of 268 swimmers. The ages of the swimmers comprise age ranges, for junior WCs (14 to 18 years), and for the senior WCs and OGs (19 to 42 years).

The dependent variables used were time and positions (finalists in junior and senior WCs and OGs), with the independent variables being gender, age, year of competition, events (50, 100, 200, 400, 800, and 1500 m), country (USA and SPA), stroke (freestyle, backstroke, breaststroke, butterfly, and individual medley), and distance event (short (50–100 m), mid (200–400 m), and long distance (800–1500 m)).

### 2.2. Procedure

The data from both databases (WC and OG) were sorted alphabetically and filtered, and all of the data were later joined in a single database, with finalist swimmers’ data in both the junior and senior categories being taken from the FINA page. Then, the final filtered database was created from the whole population of finalist swimmers from the USA and SPA competing in junior/senior WC and OGs. This study was carried out with the data obtained from the FINA page, so informed consent was not obtained, since they are public data.

### 2.3. Statistical Analysis

Data were obtained from the official website of the International Swimming Federation and added to a statistical program Excel v.2019. Firstly, swimmers from USA and SPA were ordered by competitions, with these being in the following order: junior WCs, senior WCs, and OGs. They placed themselves in this order according to the different variables. Disqualified swimmers were removed, and the final filtered database was placed in the Jamovi software (v.1.2.27).

Descriptive analysis was carried out to find out the number of junior and senior swimmers, as well as the number of swimmers who participated in each stroke and each year. Descriptive analysis was also used to find out the average time of the swimmers depending on the distance and stroke.

Then, the database was placed in R statistical software v4.1.2. Explanatory models of the z scores from age for the different distances and strokes were estimated. Quadratic regression models were developed to capture the behavior of the variable time with parabolic functions. These models were compared between Spanish and American swimmers.

The significance of the global model and the predictor variables were evaluated. In those cases, in which the values do not present a significant pattern, derived mainly for the Spanish case due to an insufficient sample size, the estimates were not considered. Likewise, the graphs of the point and the estimated parabolic curve were carried out. The level of significance was set at *p* < 0.05.

## 3. Results

Descriptive analyses showed that of the whole finalist sample (268 swimmers), 97 (36.19%) belong to the junior category and 169 (63.81%) to belong the senior category. In relation to gender in the junior category, 44.33% were men and 55.67% were women, and in the senior category, 48.54% were men and 51.46% were women. Of the 268 swimmers analyzed in the present study, there is a greater number of USA swimmers compared to SPA swimmers in the various championships.

When analyzing the variable of years of competition between 2006 and 2021, in the junior category, the year with the highest number of finalist swimmers was 2011 with 36 (37.11%). In the senior category in the Olympics, the year with the highest number of finalists was 2021 with 30 (17.54%) swimmers and in the WCs in 2007, 29 (16.96%) swimmers achieved a great difference in the number of finalists compared to the following years.

In the junior WCs, there was a greater number of finalists in the freestyle group, compared to the rest of the strokes in the three competitions in which the USA participated.

Regarding the Spanish swimmers, there was a lower participation in the finals than the USA swimmers. The highest participation in junior WCs was achieved by a total of four swimmers, separated by gender. No participation was achieved in the junior WCs for backstroke and breaststroke.

According to the test carried out, depending on the time, the USA swimmers obtained better results in the WCs and senior Olympics compared to the junior WCs. However, Spanish swimmers failed to participate in the events (50 m and 1500 m) of the Olympic Games and did not have a single participant in any event.

Of the total swimmers, only 77 (28.4%) reached junior WCs and 35 (12.97%) only reached senior WCs without having previously been in junior WC finals or the Olympics. In total, 48 (17.71%) swimmers managed to reach the finals of the Olympics. The percentage of swimmers that participated in junior WC and senior WC finals was only 4.4%, with a total of nine swimmers belonging to the USA, and three to Spain.

However, the junior WC and Olympics finalists had only five participants, all corresponding to the USA, with male (60%) and a female (40%) participation. Seven swimmers reached junior WCs, senior WCs, and Olympic Games, of which 85.6% were from the United States and 14.4% were Spanish with a single participant.

### 3.1. Results by Age, Stroke, and Country

Estimated explanatory models of z scores based on age for the different strokes and countries are shown in Table 1.

The peak performance in freestyle was 29.81 and 28.91 years old, 23.25 and 24.52 in backstroke, 22.75 and 32.54 in butterfly, and 31.02 and 26.30 in an individual medley for Spain and USA, respectively. There were not enough data in our sample to show a significant result for breaststroke.

The characteristics of the results obtained in sports competitions by age (peak performance) were examined (Table 1).

**Figure 1:**
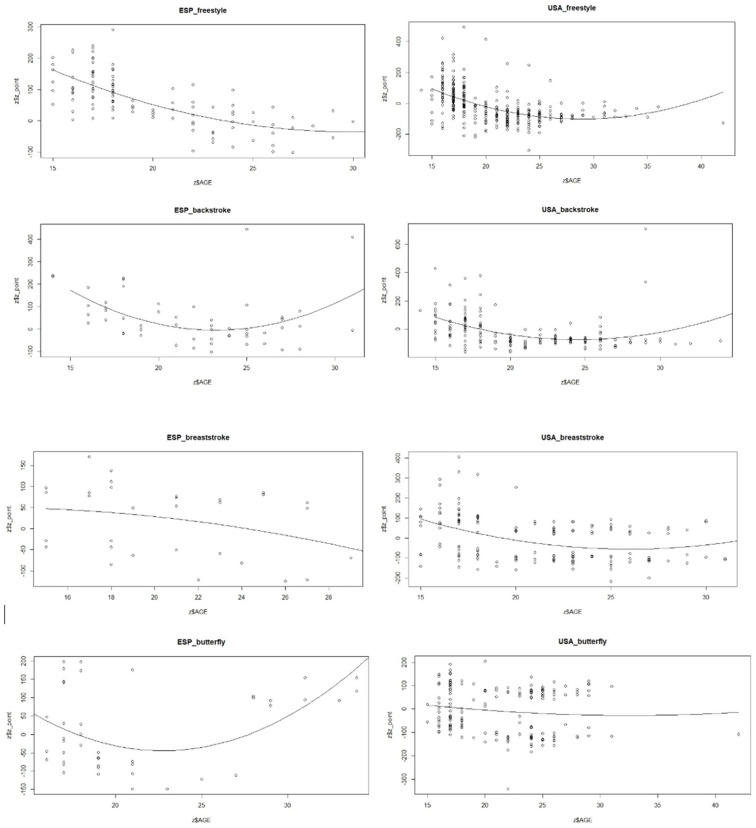
Model curvature by age, style, and country.

The general and predictor variables’ significances are also shown in Table 2. Optimal age (peak performance) as well as the curvature of the model, which measures the fastness with which they progress to said peak from early ages (the higher the value, the more intensity in the progress), are shown in Figure 1. The most remarkable results are shown in backstroke for both SPA and USA and for butterfly in SPA.

### 3.2. Results by Modality (Short/Medium/Long Distance)

Estimated explanatory models of z scores based on age for the different modalities and countries are shown in Table 2.

The peak performance in short distances is 24.66 and 29.95 years old, 23.90 and 24.65 in medium distances, and 31.76 and 26.99 in long distances for Spain and USA, respectively.

**Figure 2:**
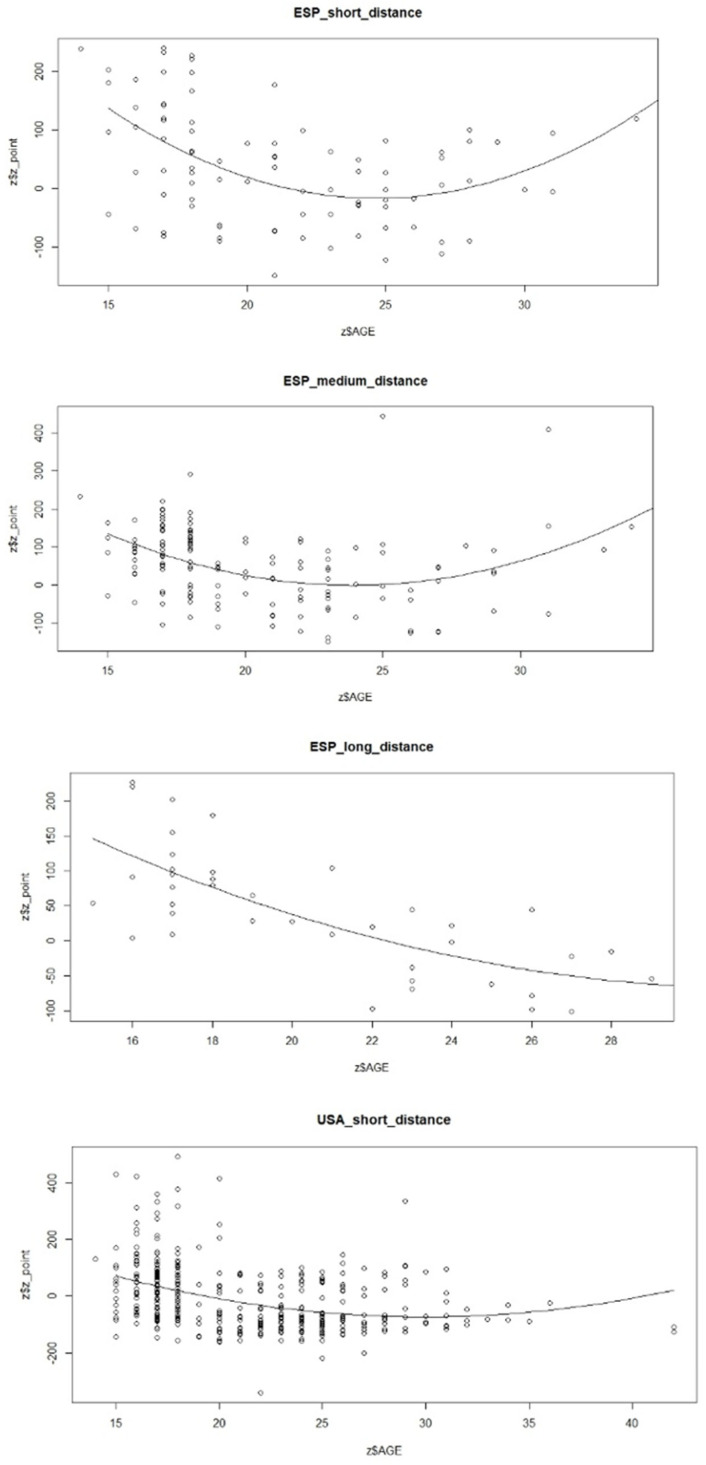
Age peak performance by modality for Spain and USA.

Also, comparative charts of age peak performance by modality for SPA and USA are shown in Figure 2.

## 4. Discussion

The main objective of this study was to analyze the effect of early specialization in swimming of two different early specialization systems (USA vs. SPA). In addition, another objective was to observe the general patterns of the success of SPA and USA swimmers participating in the Olympic Games and World Championships between the years 2006 and 2021 for all strokes and distances.

The main finding was that of the total sample of swimmers, the number of swimmers who reach the final in senior WCs and OGs, having previously participated in junior WCs, is quite scarce (seven swimmers). A third of the first group of athletes who were selected in the junior category, and in the senior category, were consolidated among the best swimmers [9]. Being a finalist in the junior WCs does not indicate that it is the same way in the senior WCs [13] since many of the swimmers selected in the junior category do not manage to reach the senior category [11]. This is due to the early specialization that some swimmers have in the junior stages, with only a third of the athletes selected in the junior category reaching the senior category, consolidating themselves among the best [9]. Increased consolidation is one of the possible causes of injuries [17], without knowing the age at which there may be a greater risk, and since swimmers perform intense training, this is also a possible cause of injury [18]. It is advisable to play several sports at an early age, and also in adolescence, to specialize in a sport to obtain greater success and minimize injuries, psychological stress, and physical exhaustion [19]. These data are related to the results of this study since 35 of the swimmers reached the finals of the senior WCs and 48 reached the finals in the WCs.

In terms of time, USA swimmers swim faster in major tournaments [15]. This shows similarity with the results obtained since the USA obtained better results than Spain in the junior, senior, and Olympic WCs. Other studies found similar results [20], reaching the conclusion that finalist swimmers of senior WCs and OGs obtained a time in swimming speed that increased in both men and women, in the different events and strokes analyzed. Improvements in the female gender in the long-distance events and in the male gender in the short-distance events over the years were obtained, with the time of those who achieved a podium being 0.6% greater than the rest of the finalists [21]. This shows a relation to the results obtained since both USA and Spain obtained better times in senior WCs and OGs than in junior WCs. An increase in performance was shown over the years in all genders in all the distances and strokes analyzed for senior WCs and OGs [1]. Above all, the participation of women increased in all strokes over the years [22]. These results are similar to the results obtained in our study, where a greater number of women was observed in almost all competitions in both countries over the years.

On the other hand, OG finalist swimmers were 0.5% faster than the senior WC swimmers [23]. The USA has many inhabitants, as well as purchasing power, first-class sports facilities, and well-trained coaches [24]. The results of this study showed the difference between the two countries, since the USA manages to have a greater number of swimmers in the finals of the junior and senior WCs, as well as in the OGs, both in the junior and senior categories. This situation could be due to the great involvement of the country in the training and accompaniment of elite swimmers from an early age.

Help in university studies or even from earlier stages to combine high-level sports with training is a matter that should be considered in the rest of the countries and in this specific case in Spain to give the opportunity to a greater number of talented swimmers to participate in these high-level competitions. In this way, swimmers, federations, and the government itself would obtain the rewards for the efforts of both money and time that are provided in the sports world.

## 5. Conclusions

Most of the finalist swimmers of the junior WCs do not manage to reach the finals of the senior WCs and OGs, with only seven swimmers from both countries reaching the three finals of junior WCs, senior WCs, and OGs between the years 2006 and 2021 for all strokes and distances. Therefore, early participation in high-level competition is not an indicator of further success for USA and SPA swimmers.

In total, 85.6% of the swimmers participated in the representation of the USA and 14.40% represented Spain. A larger population of the USA is one of the possible causes of this representation, as well as a greater purchasing power of the country, and scholarships that athletes receive from an early age.

The main differences in patterns of success between SPA and USA are the earlier participation of USA swimmers in high-level competitions as well as the greater number of participants for all the strokes, events, genders, and competitions. Age peak performance analyses showed that younger swimmers participated in short-distance events, backstroke, and butterfly while older swimmers participated in long-distance events and individual medley for Spanish swimmers. For USA swimmers, older swimmers participated in butterfly and short-distance events.

## Figures and Tables

**Table 1:** Estimated explanatory models of z scores based on age for the different countries and styles.

	SPAIN		USA	
**FREESTYLE**				
	**Estimate**	**Pr (>|t|)**	**Estimate**	**Pr (>|t|)**
**(Intercept)**	764.81	6.32 × 10^−5^ ***	750.07	1.16 × 10^−15^ ***
**AGE**	−53.64	0.00 **	−58.88	6.22 × 10^−13^ ***
**I (AGE^2^)**	0.89	0.031 *	1.01	3.90 × 10^−9^ ***
**Global significance**	F-statistic: 53.5 on 2 and 104 DF	<2.2 × 10^−16^	F-statistic: 70.94 on 2 and 337 DF	<2.2 × 10^−16^
**BACKSTROKE**				
**(Intercept)**	1403.35	0.00 ***	978.80	1.45 × 10^−6^ ***
**AGE**	−121.12	0.00 ***	−85.89	5.76 × 10^−6^ ***
**I (AGE^2^)**	2.60	0.00 ***	1.75	4.02 × 10^−5^ ***
**Global significance**	F-statistic: 8.621 on 2 and 51 DF	0.00	F-statistic: 19.46 on 2 and 163 DF	2.635 × 10^−8^
**BREASTSTROKE**				
**(Intercept)**	6.61	0.98	786.62	0.000489 ***
**AGE**	7.61	0.85	−64.92	0.002059 **
**I (AGE^2^)**	−0.32	0.74	1.25	0.008862 **
**Global significance**	F-statistic: 1.505 on 2 and 28 DF	0.23	F-statistic: 17.43 on 2 and 161 DF	1.402 × 10^−7^
**BUTTERFLY**				
**(Intercept)**	875.26	0.02 *	125.01	0.330
**AGE**	−80.81	0.01 *	−9.36	0.405
**I (AGE^2^)**	1.77	0.00 **	0.14	0.551
**Global significance**	F-statistic: 5.673 on 2 and 39 DF	0.00	F-statistic: 1.431 on 2 and 164 DF	0.24
**INDIVIDUAL MEDLEY**				
**(Intercept)**	669.64	0.04 *	1202.76	1.01 × 10^−9^ ***
**AGE**	−47.31	0.11	−100.12	2.75 × 10^−8^ ***
**I (AGE^2^)**	0.76	0.25	1.90	1.89 × 10^−6^ ***
**Global significance**	F-statistic: 11.28 on 2 and 35 DF	0.00	F-statistic: 60.52 on 2 and 113 DF	<2.2 × 10^−16^

Signif. codes: 0 ***; 0.001 **; 0.01 *.

**Table 2:** Estimate explanatory models of z scores based on age for the different modalities and countries.

	SPAIN		USA	
**SHORT DISTANCE**				
	**Estimate**	**Pr (>|t|)**	**Estimate**	**Pr (>|t|)**
**(Intercept)**	980.6717	4.21 × 10^−6^ ***	510.3470	7.01 × 10^−9^ ***
**AGE**	−80.8686	2.78 × 10^−5^ ***	−38.9523	3.31 × 10^−7^ ***
**I (AGE^2^)**	1.6392	0.00011 ***	0.6502	4.70 × 10^−5^ ***
**Global significance**	F-statistic: 15.03 on 2 and 86 DF	2.528 × 10^−6^	F-statistic: 37.86 on 2 and 394 DF	9.105 × 10^−16^
**MEDIUM DISTANCE**				
**(Intercept)**	978.8874	2.20 × 10^−7^ ***	1010.5717	3.20 × 10^−15^ ***
**AGE**	−81.9326	1.75 × 10^−6^ ***	−87.3971	4.25 × 10^−13^ ***
**I (AGE^2^)**	1.7140	5.35 × 10^−6^ ***	1.7725	1.31 × 10^−10^ ***
**Global significance**	F-statistic: 14.59 on 2 and 141 DF	1.739 × 10^−6^	F-statistic: 68.47 on 2 and 467 DF	<2.2 × 10^−16^
**LONG DISTANCE**				
**(Intercept)**	696.8275	0.043 *	890.4714	0.00282 **
**AGE**	−48.1154	0.138	−74.6555	0.00998 **
**I (AGE^2^)**	0.7573	0.310	1.3828	0.04422 *
**Global significance**	F-statistic: 21.18 on 2 and 36 DF	8.309 × 10^−7^	F-statistic: 23.73 on 2 and 83 DF	7.05 × 10^−9^

Signif. codes: 0 ***; 0.001 **; 0.01 *.

## Data Availability

This study was carried out with the data obtained from the FINA page, so informed consent was not obtained, since they are public data.
